# Iatrogenic constipation from barium blockade: A case report

**DOI:** 10.1002/ccr3.2280

**Published:** 2019-07-10

**Authors:** Shajadi Pathan, Taras Benzar, Samip Master, Prakash Peddi

**Affiliations:** ^1^ Feist-Weiller Cancer Center Louisiana State University Shreveport Louisiana; ^2^ Department of Internal Medicine Louisiana State University Shreveport Louisiana

**Keywords:** barium blockade, barolith, constipation, stercoral colitis

## Abstract

Barolith, a mixture of inspissated barium and feces, is a rare complication of barium‐contrast studies that lead to intestinal obstruction. With the high morbidity associated with barolith impaction, we recommend that physicians be more aware of complications, increase prompt diagnosis, and initiation of laxatives once discovered.

## 1 INTRODUCTION

Overflow incontinence is a well‐known complication that occurs from chronic constipation. These patients develop paradoxical small volume stool leakage, which often times can be misinterpreted as diarrhea. This case report describes a 64‐year‐old woman who presented with the chief complaint of “diarrhea and abdominal cramps” and was diagnosed with overflow incontinence secondary to intestinal obstruction from inspissated barium and feces forming a “barolith” which was a result of barium contrast roentgenography performed 9 months prior to the onset of presentation. No underlying colonic pathology was identified.

## 2 CASE HISTORY

We report a case of a 64‐year‐old woman presented to the emergency department with an acute onset of diarrhea and cramping abdominal pain for two days. She was in her normal state of health until 9 days prior to the admission, when she became increasingly constipated. This was later followed by the onset of liquid stools at hourly intervals.

Her review of systems was positive for loss of weight of 10lbs over 8 weeks and was negative for any associated fever, chills, nausea, vomiting, melena, or hematochezia. On examination, she appeared cachectic and malnourished with dry mucous membranes, soft and nontender abdomen with hyperactive bowel sounds. Rectal exam revealed normal tone and no sign of appreciable fecal impaction.

**Figure 1:**
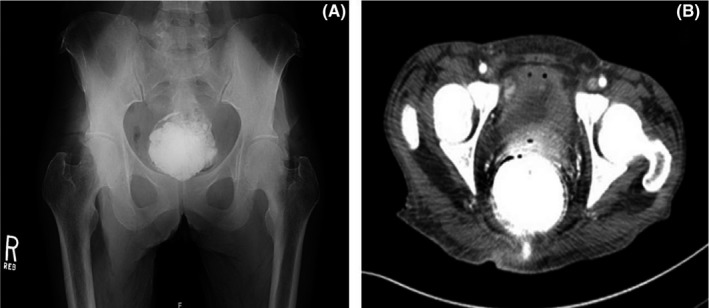
A, the abdominal x‐ray showed a large radiopaque abnormality, measuring about 7.5 × 6.3 cm, suggesting the possibility of retained barium. B, CT abdomen and pelvis with oral and intravenous contrast were consistent with barolith fecal impaction with evidence of stercoral colitis

Her past medical history was significant for well‐controlled hypertension and stage III gastric cancer. At the time of initial diagnosis, staging computed tomography (CT) scan with intravenous (IV) and oral contrast revealed peri‐gastric lymphadenopathy and focal thickening of the stomach wall. To the best of our knowledge, no further barium studies had been performed. She later underwent total gastrectomy with D2 nodal dissection with esophagojejunostomy. The patient was subsequently started on adjuvant chemotherapy. Three weeks after completion of her chemotherapy, the patient presented with the above symptoms. The patient underwent abdominal plain film imaging (Figure 1A) along with CT abdomen and pelvis (Figure 1B) revealing large radiopaque abnormality consistent with barolith. Initial management consisted of administration of mineral oil enema and a trial of digital disimpaction. As both failed, fecal disimpaction under general anesthesia was undertaken. The patient tolerated this procedure well and soon resumed normal bowel function.

## 3 DISCUSSION

In the geriatric population, the association between fecal incontinence and constipation has long been recognized with fecal impaction accepted as being the most common cause of involuntary stool loss.^1^ Prolonged retention of stool in the rectum, perhaps secondary to incomplete evacuation during defecation, physical immobility, inadequate diet, and water intake, associated neurological and metabolic disorders, or use of anti‐motility drugs, can lead to fecal impaction.^2^, ^3^, ^4^ This may result in overflow incontinence, which causes liquid stool to seep around the fecal bolus.^5^ The presence of an impacted mass will also stimulate the secretion of large volumes of mucus, which will further aggravate the problem.^5^, ^6^

The pathophysiology of constipation and stool incontinence is multifactorial. The three major postulated mechanisms are as follows: (a) overflow incontinence from stool impaction, (b) involuntary, usually postdefecation leakage, due to retention of stool in the rectum, and (c) secondary to generalized pelvic floor weakness.^7^

Barium sulfate is the sulfate salt of barium which is an alkaline divalent metal. Barium sulfate, a water‐insoluble compound, is used in routine radiological procedures as a contrast medium. It not only remains the gold standard technique for imaging for fine mucosal details but also remains the best technique for demonstrating general gastrointestinal tract configuration and caliber.^8^ Complete inertness of barium, its low cost, and its effectiveness have made barium the most widely used oral contrast agent. Once barium is swallowed, it is shown to be cleared from the colon within a week in 57% of patients and completely cleared within 4 weeks in most patients.^9^

Baroliths refer to inspissated barium and fecal admixture which is formed after oral or rectal administration of barium sulfate contrast.^10^ Baroliths are often asymptomatic, but as the volume of the inspissated mass increases, it is associated with abdominal pain, nausea, emesis, severe constipation, bowel obstruction, or rarely barium peritonitis from bowel perforation.^11^ Baroliths causing colorectal obstruction, colon wall necrosis, and perforation, appendicitis, megarectum, abdominal compartment syndrome, have been reported in the literature.^12^ Constipation is the most common symptom that patients present after a barium study. The interval between a barium study and symptomatic manifestation of barolith may range from 2 days to 2 years.^13^

In a systemic review of 31 cases by Kurer et al, factors contributing to a barolith formation were noted to include the history of abdominal surgeries, gut immobility, underlying pathology, and the patient's age, state of hydration, and diet.^14^ The other factors which are associated with barolith formation include inappropriate dilution of contrast medium that contributes to increased sedimentation, intake of solid foods before contrast, and lack of use of laxative treatment after barium studies to reduce the incidence of colonic retention of barium sulfate.^15^ Among the reported cases, the most common site for barolith impaction in the reported cases was the descending colon. In the current case, constipation and opioid use were contributing factors.

Currently, there are no recommended guidelines for the treatment of baroliths. For patients without clear indications for emergency surgery, treatment modalities range from manual disimpaction, enemas, and lactulose. In current literature, we identified three cases where barolith was not dislodged by conservative methods and colonoscopic dissolution was attempted and was successful in all of them.^14^

In the presence of the above‐mentioned risk factors, a follow‐up imaging 2‐3 days later is recommended and, if barium residue remains, a laxative should be administered immediately.

Our patient had overflow incontinence which was initially interpreted as diarrhea, which later proved to be fecal impaction as was evident from further imaging. Reduced colonic motility from chronic opiates may have contributed to the development of barolith 9 months after initial administration of barium contrast. This case highlights the importance of considering overflow incontinence in the differential diagnosis of patients who have risk factors for chronic constipation and who present with small volume diarrhea and abdominal cramps. Chronic constipation and overflow incontinence are not uncommon in cancer patients who are on opiates for pain control. Complications from these may lead to increased emergency room visits, hospitalizations, and delays in chemotherapy.

## CONFLICT OF INTEREST

None declared.

## AUTHOR CONTRIBUTION

PS and BT: wrote the case report with support from MS and PP, who also supervised the project. All authors read and approved the final manuscript.
